# Peroxiredoxin I is important for cancer-cell survival in Ras-induced hepatic tumorigenesis

**DOI:** 10.18632/oncotarget.11172

**Published:** 2016-08-10

**Authors:** Bing Han, Hye-Jun Shin, In Seon Bak, Yesol Bak, Ye-Lin Jeong, Taeho Kwon, Young-Ho Park, Hu-Nan Sun, Cheol-Hee Kim, Dae-Yeul Yu

**Affiliations:** ^1^ Genome Editing Research Center, Korea Research Institute of Bioscience and Biotechnology, Daejeon, 305-806, Korea; ^2^ Department of Biology, Chungnam National University, Daejeon, 305-764, Korea; ^3^ Department of Toxicology Evaluation, Graduate School of Preclinical Laboratory Science, Konyang University, Daejeon, 363-700, Korea; ^4^ Department of Bioscience and Biotechnology, Bio/Molecular Informatics Center, Konkuk University, Seoul, 143-701, Korea; ^5^ Department of Animal Biosystem Sciences, Chungnam National University, Daejeon, 305-764, Korea; ^6^ College of Life Science and Biotechnology, Heilongjiang Bayi Agricultural University, Daqing, 163319, China

**Keywords:** peroxiredoxin I, H-ras^G12V^, hepatic tumorigenesis, reactive oxygen species, gene regulation

## Abstract

Peroxiredoxin I (Prx I), an antioxidant enzyme, has multiple functions in human cancer. However, the role of Prx I in hepatic tumorigenesis has not been characterized. Here we investigated the relevance and underlying mechanism of Prx I in hepatic tumorigenesis. Prx I increased in tumors of hepatocellular carcinoma (HCC) patients that aligned with overexpression of oncogenic H-ras. Prx I also increased in H-ras^G12V^ transfected HCC cells and liver tumors of H-ras^G12V^ transgenic (Tg) mice, indicating that Prx I may be involved in Ras-induced hepatic tumorigenesis. When Prx I was knocked down or deleted in HCC-H-ras^G12V^ cells or H-ras^G12V^ Tg mice, cell colony or tumor formation was significantly reduced that was associated with downregulation of pERK pathway as well as increased intracellular reactive oxygen species (ROS) induced DNA damage and cell death. Overexpressing Prx I markedly increased Ras downstream pERK/FoxM1/Nrf2 signaling pathway and inhibited oxidative damage in HCC cells and H-ras^G12V^ Tg mice. In this study, we found Nrf2 was transcriptionally activated by FoxM1, and Prx I was activated by the H-ras^G12V^/pERK/FoxM1/Nrf2 pathway and suppressed ROS-induced hepatic cancer-cell death along with formation of a positive feedback loop with Ras/ERK/FoxM1/Nrf2 to promote hepatic tumorigenesis.

## INTRODUCTION

The second leading cause of cancer death worldwide and the sixth most common cancer in the world is hepatocellular carcinoma (HCC). The age-standardized rate per 100,000 ranges from 13.4 (Guinea-Bissau) to 78.1 (Mongolia). Hepatitis B or C virus, alcoholic drinks, and aflatoxins are factors causing HCC; oxidative stress, inflammation, and immune response are important for hepatic tumor growth [1]. In addition, oxidative stress (reactive oxygen species)-induced cellular DNA damage leads to gene mutation and activation of multiple signaling pathways that promote many aspects of hepatocellular-tumor development and progression [2].

In the liver-tumor microenvironment, balancing cellular ROS is important to tumor-cell maintenance. Low doses of ROS upregulate mRNA levels of cyclins to stimulate cell proliferation, promoting tumor growth; however, disproportionately increased cellular ROS induces cancer-cell cycle arrest, senescence, and apoptosis. Therefore, ROS scavengers and antioxidant enzymes are essential to tumorigenesis. Depletion of antioxidant proteins from cells increases cellular ROS causing cell apoptosis, which is one method of cancer therapy [3–5].

Peroxiredoxins (Prxs) are thioredoxin peroxidases that catalyze hydrogen peroxide, organic hydroperoxides, and peroxynitrite [6, 7]. They are important ROS scavengers. Mammalian cells have six isoforms of Prx, divided into three subfamilies based on catalytic cysteine (Cys) residue. Prx I belongs to 2-Cys Prxs. Recent evidence suggests that Prx I intervenes cell signaling by activating several signaling proteins to regulate cell proliferation, differentiation, and apoptosis [8–10]. Moreover, the role of Prx I in human cancer is still controversial and the different role of Prx I in human cancer depends on the specific cellular context. In several malignant cancers like lung cancer, it acts as a tumor suppressor. In K-ras-driven lung tumorigenesis mouse models, Prx I suppresses K-ras-induced ROS/ERK/cyclin D1 signaling pathways to inhibit lung tumorigenesis [11]. In contrast, in breast cancer and prostate cancer, it acts as a pro-oncogenic factor. Expression of Prx I in breast cancer facilitates cancer-cell survival from oxidative stress-induced cell death [12]; in prostate cancer, Prx I binds with TLR4 and thus regulates growth factor VEGF expression to promote prostate carcinogenesis [13].

Ras-family genes (H-ras, K-ras, and N-ras) are the most frequently altered human cancers [14–16]. H-ras mutation was found in HCC patients and contributed to tumor development [17–20]. In normal mice and HCC cell lines, the mutant form of H-ras^G12V^ is not present. Our laboratory generated hepatic carcinoma H-ras^G12V^mouse model and stable HCC-H-ras^G12V^ cell lines by transfecting mutant form H-ras^G12V^ into mouse embryo and HCC cell lines. In Tg mice and HCC-H-ras^G12V^ cell lines, mutant oncogenic Ras leads to elevated intracellular ROS levels and activation of the ERK/FoxM1/cyclin D1 pathway to promote hepatic tumorigenesis [21].

Previously reported that Prx I and mutant form of H-ras have well correlated with HCC, respectively [17–20, 27]. To determine the relationship between Prx I and H-ras induced Hepatic tumorigenesis, we generated H-ras^G12V^/Prx I^−/−^ double mutant mice and analyzed phenotypes of the mice. We investigated molecular events by performing knockdown or overexpression of Prx I in HCC-H-ras^G12V^ cell lines. Our results illustrated that Prx I suppresses ROS-induced apoptosis and positively regulates the Ras/ERK/FoxM1/Nrf2 pathway to promote hepatocacinogenesis. Therefore, Prx I, a novel target ROS, plays a role in hepatic cancer-cell survival, and will be a potential therapeutic target for HCC prevention and treatment.

## RESULTS

### Prx I was overexpressed in HCC patient tissues, H-ras^G12V^-HCC cells, and the H-ras^G12V^ Tg mice hepatic-tumor region

**Figure 1:**
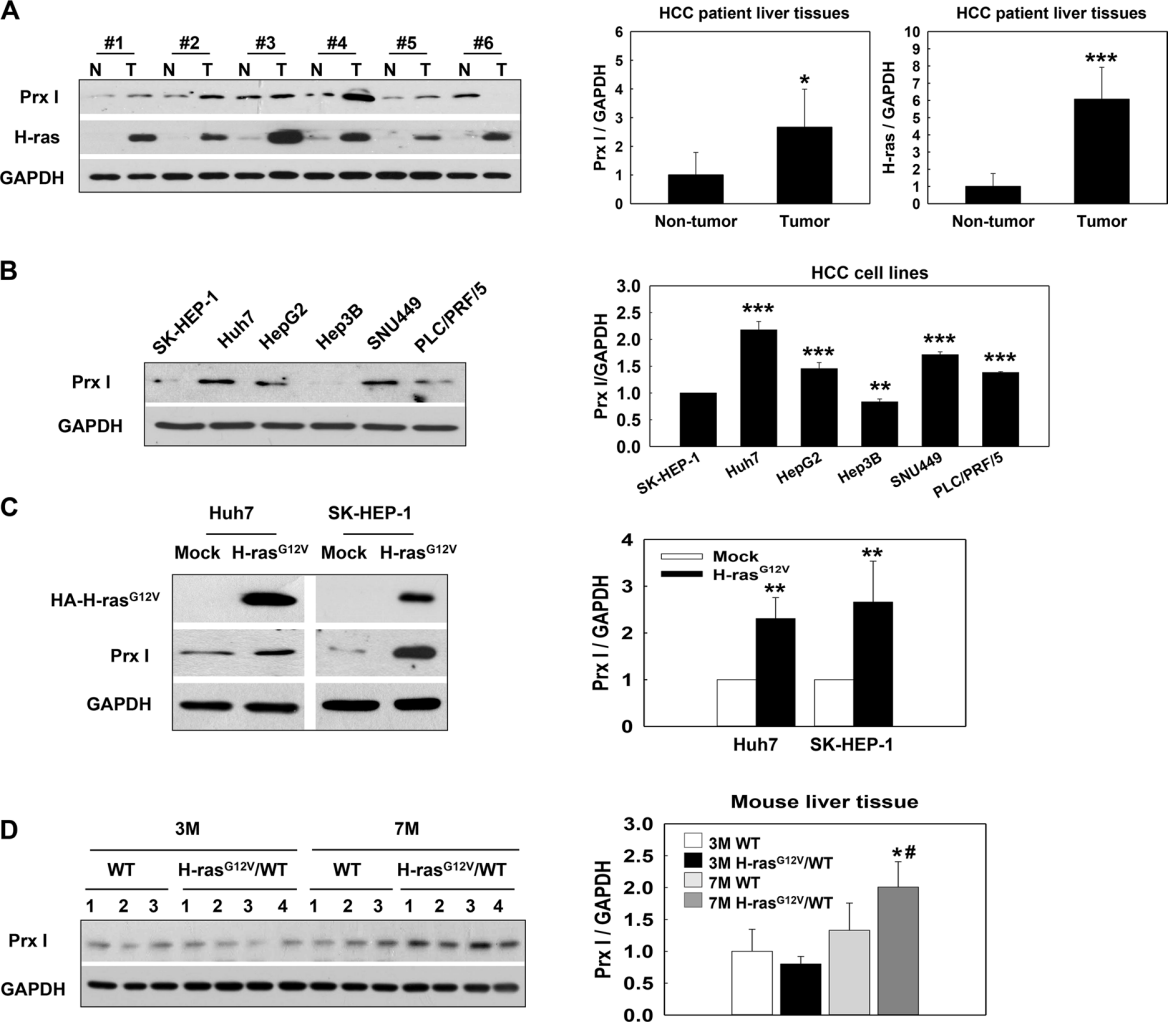
Prx I overexpression linked with oncogenic Ras expression in HCC patient tissues, HCC-H-ras^G12V^ cells, and the H-ras^G12V^ Tg mice hepatic-tumor region. (**A**) HCC patient-tumor region (T) and non-tumor region (N) were analyzed by Western blotting with Prx I, H-ras, and GAPDH antibodies. GAPDH was used as a loading control. Samples 1, 3, and 6 were from hepatocellular carcinoma patients' liver tissues. Samples 2, 4, and 5 were from hepatitis B virus patients' liver tissues. *p < 0.05, ***p < 0.001 compared to non-tumor. (**B**) Western blotting analysis of Prx I expression in HCC cells. **p < 0.01, ***p < 0.001 compared to SK-HEP-1. (**C**) The expression level of Prx I in Huh7 and SK-HEP-1 stable cell lines transfected by the pCAG-HA (Mock) or the pCAG-HA-H-ras^G12V^ vector. HA is a tag of H-ras^G12V^ protein. **p < 0.01, compared to Mock cells. (**D**) Using immunoblotting to detect Prx I expression in C57BL/6 wild type (WT) or H-ras^G12V^/WT Tg mice liver tissues. *p < 0.05, compared to 3M H-ras^G12V^/WT and ^#^*p* < 0.05 compared to 7 M WT. The data were repeated in at least three separate experiments.

**Figure 2:**
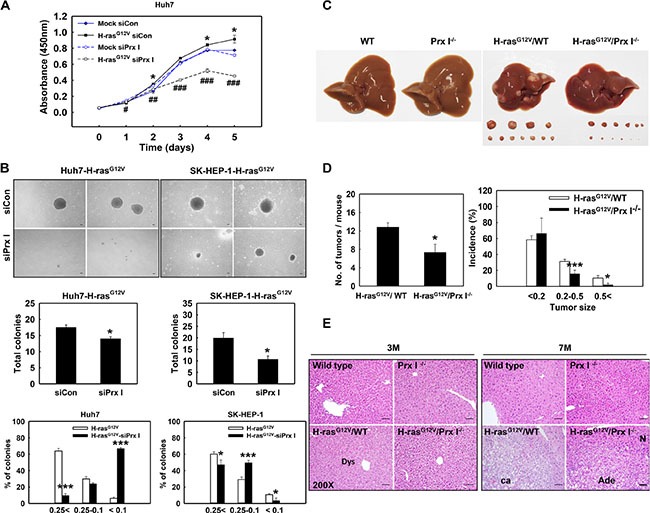
Prx I promoted Ras-induced hepatocarcinogenesis (**A**) Huh7-Mock and Huh7-H-ras^G12V^ cells were transiently transfected with scramble siRNA (siCon) or siPrx I. After incubation, cell proliferation was determined by CCK8 assay at the indicated time. *p < 0.05 compared to Huh7-Mock-siCon cells and ^#^*p* < 0.05, ^##^*p* < 0.01, ^###^*p* < 0.001 compared to Huh7-H-ras^G12V^-siCon cells. (**B**) Anchorage-independent growth in soft agar were performed in Huh7-H-ras^G12V^ and SK-HEP-1-H-ras^G12V^ cells after transfected with siRNA (scramble or Prx I). Cell morphologies were observed under an inverted-phase contrast microscope at × 40 magnification. Scale bars, 100 μm. The number of colonies was determined by counting duplicated plates, *p < 0.05 compared to siCon. The diameters of the colonies were 0.25 mm <, 0.25–0.1 mm, and < 0.1 mm, *p < 0.05, ***p < 0.001 compared to H-ras^G12V^. (**C**) The gross appearance of WT, Prx I^−/−^, H-ras^G12V^/WT, and H-ras^G12V^/Prx I^−/−^ mice liver at 7 months. (**D**) Tumor number and tumor size were measured at the age of 7 months H-ras^G12V^/WT (*n* = 6) and H-ras^G12V^/Prx I^−/−^ (*n* = 7) mice-liver region. Tumor size; long × short diameter, cm2 (< 0.2 cm^2^, 0.2–0.5 cm^2^ and 0.5 < cm^2^). *p < 0.05, ***p < 0.001. (**E**) (H&E) staining of livers at 3 months and 7 months of WT, Prx I^−/−^, H-ras^G12V^/WT, and H-ras^G12V^/Prx I^−/−^ mice. Magnification, 200 X. Scale bars, 100 μm. The data were repeated in at least three separate experiments and presented as mean ± SD.

Prx I and H-ras were expressed significantly higher in the tumor region of HCC patients than the non-tumor region (Figure 1A). In two distinct Prx I expression pattern HCC cell lines, Huh7 and SK-HEP-1 (Figure 1B), after v-HA-Ras-vector transfection, the Prx I protein level significantly increased in both cells (Figure 1C). At the age of 3 months H-ras^G12V^ Tg mice liver showed dysplasia (Dys) and Prx I expression was no different compared with wild type (WT). However, at 7 months H-ras^G12V^ Tg mouse livers showed carcinoma and Prx I expression increased significantly in the tumor region (Figures 1D and 2E). Consistently, these results indicated that Prx I expression can be regulated by oncogenic Ras, and correlated with tumor progression.

### Prx I promoted Ras-induced hepatocarcinogenesis

H-ras^G12V^ transfected HCC cells grew faster than HCC-Mock cells (Figure 2A); H-ras^G12V^ overexpression significantly increased anchorage-independent growth in HCC cells (Figure 2B); H-ras^G12V^ Tg mice at the age of 7 months showed hepatic carcinoma in the liver region (Figure 2C and 2E). To investigate the role of Prx I in H-ras^G12V^-induced hepatocarcinogenesis, we knocked down Prx I in HCC-H-ras^G12V^ cells by treating with siPrx I, and generated H-ras^G12V^/Prx I^−/−^ double mutant mice. CCK8 assay data showed that siPrx I significantly decreased the growth speed of HCC-H-ras^G12V^ cells from the 3rd day, dramatically suppressed cell proliferation (Figure 2A). Consistently, soft-agar assay results showed that knockdown of Prx I in HCC-H-ras^G12V^ cells significantly suppressed colony formation (Figure 2B). Tumor numbers of H-ras^G12V^/Prx I^−/−^ double mutant mice at 7 months decreased significantly; tumor size was markedly smaller than in H-ras^G12V^/WT mice (Figure 2C and 2D). The histological data showed that deletion of Prx I significantly suppressed H-ras^G12V^-mediated hepatic tumorigeneisis (Figure 2E). These results suggest that Prx I promotes oncogenic Ras-induced hepatocarcinogenesis.

### Prx I modulated tumorigenesis through positive regulation of pERK and cyclin D1 expression

**Figure 3:**
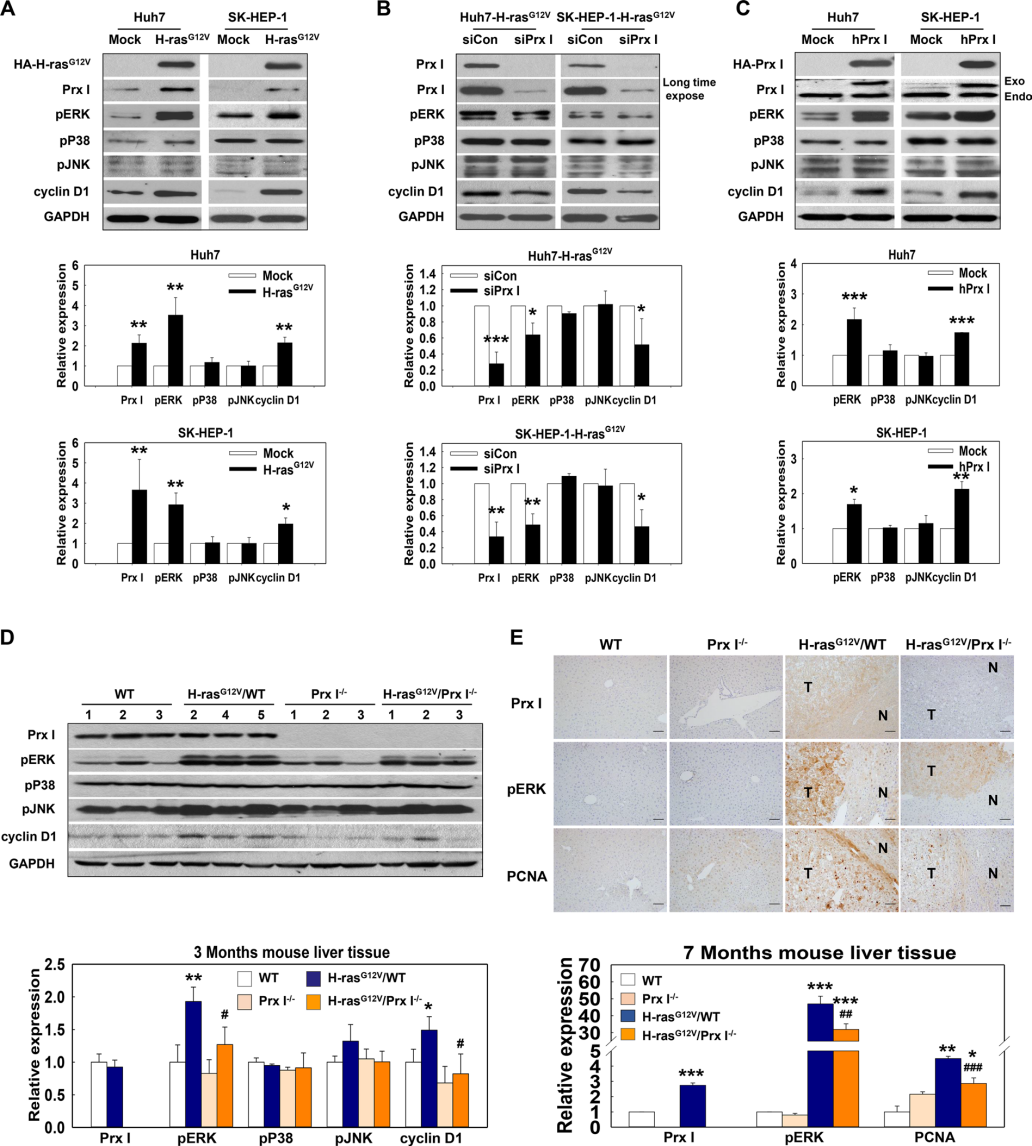
Prx I regulated the Ras-activation pathway (**A**–**D**) Protein expression levels of Prx I, cyclin D1, and GAPDH as well as phosphorylation levels of ERK, P38, and JNK were analyzed in HCC-H-ras^G12V^ cells (A), siPrx I-transfected HCC-H-ras^G12V^ cells (B), pCAGGS-hPrx I-HA-neo-transfected Prx I-HCC cells (C), and 3 months WT, Prx I^−/−^, H-ras^G12V^/WT, and H-ras^G12V^/Prx I^−/−^ mice-liver tissues (D). In (A, B and C), *p < 0.05, **p < 0.01, ***p < 0.001 compared to Mock, in (D), *p < 0.05, **p < 0.01 compared to WT and ^#^*p* < 0.05 compared to H-ras^G12V^/WT. (**E**) Immunohistochemical staining of Prx I, PCNA and pERK at 7 months WT, Prx I^−/−^, H-ras^G12V^/WT, and H-ras^G12V^/Prx I^−/−^ mice-liver tissues. Magnification, 200 X. Scale bars, 100 μm. *p < 0.05, **p < 0.01, ***p < 0.001 compared to WT and ^##^*p* < 0.01, ^###^*p* < 0.001 compared to H-ras^G12V^/WT. HCC cells; Huh7 and SK-HEP-1 cells. In C, HA-Prx I and Exo indicate the transfected Prx I expression; Endo indicates the intracellular endogeneous Prx I. The data were repeated in at least three separate experiments.

Western blotting data showed that pERK and cyclin D1 were more highly expressed in HCC-H-ras^G12V^ cells and liver tissues of H-ras^G12V^ Tg mice at 3 months than in controls (Figure 3A and 3D). Immunohistochemical data of liver tissues of H-ras^G12V^ Tg mice at 7 months also showed consistent results (Figure 3E). To determine the underlying mechanism of Prx I function in the Ras/ERK/cyclin D1 signaling pathway, we performed knockdown of Prx I in HCC-H-ras^G12V^ cells by siRNA. Western blotting results showed that reduced Prx I protein levels significantly suppressed activation of the pERK/cyclin D1 signaling pathway (Figure 3B). Consistently, null Prx I dramatically down-regulated pERK and cyclin D1 levels in the H-ras^G12V^ Tg mice liver region, and no difference emerged between WT and Prx I^−/−^ mice (Figure 3D and 3E). To confirm regulation of Prx I on pERK and cyclin D1 expression, we overexpressed Prx I in Huh7 and SK-HEP-1 cells. Our results demonstrated that Prx I overexpression can significantly up-regulate pERK and cyclin D1 expression in both cells (Figure 3C). Collectively, these results suggest that Prx I positively affects activation of H-ras^G12V^-induced pERK and cyclin D1 expression to promote hepatic-tumor growth.

### Prx I is needed for tumor-cell survival by inhibiting ROS-induced cell death

**Figure 4:**
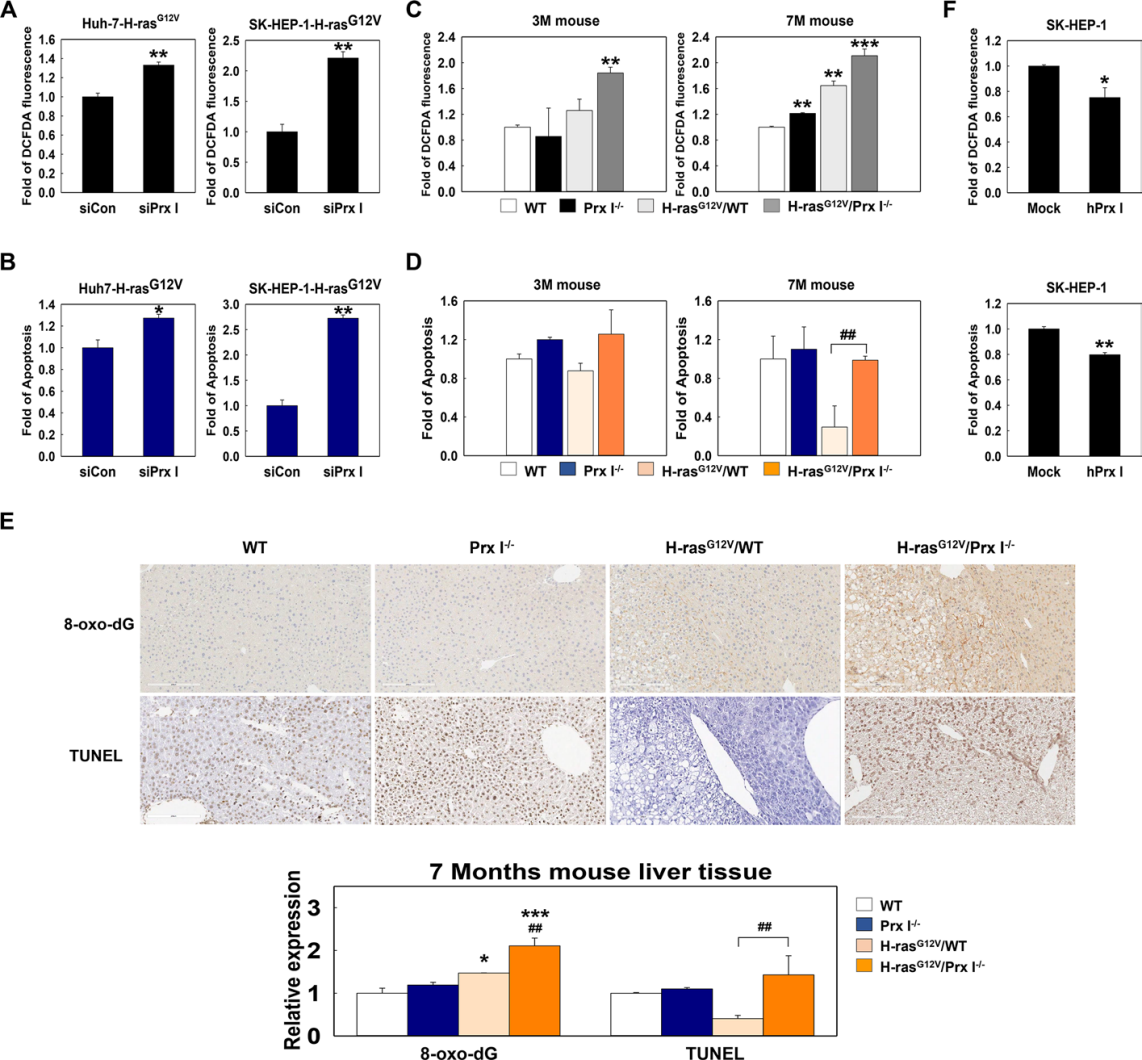
Prx I inhibited ROS induced tumor cell death (**A** and **B**) Using flow cytometry to determine intracellular ROS (A) and cell apoptosis (B) in siRNA (scramble or Prx I)-transfected HCC-H-ras^G12V^ cell lines. *p < 0.05, **p < 0.01 compared to siCon. (**C** and **D**) ROS (C) and cell apoptosis (D) level at 3 months (left panels) and 7 months (right panels) WT, Prx I^−/−^, H-ras^G12V^/WT, and H-ras^G12V^/Prx I^−/−^ mice-liver tissues were determined by flow cytometry. **p < 0.01, ***p < 0.001 compared to WT mice and ^##^*p* < 0.01 compared to H-ras^G12V^/WT mice. (**E**) Paraffin-embedded liver specimens at 7 months WT, Prx I^−/−^, H-ras^G12V^/WT, and H-ras^G12V^/Prx I^−/−^ mice were stained with 8-oxo-dG antibody and the TUNEL assay kit to detect ROS-induced DNA damage and cell death, respectively. Magnification, 200 X. Scale bars, 200 μm. *p < 0.05, ***p < 0.001 compared to WT and ^##^*p* < 0.01 compared to H-ras^G12V^/WT. (**F**) Intracellular ROS and apoptosis levels in SK-HEP-1-Mock cells and Prx I-SK-HEP-1 cells (SK-HEP-1-hPrx I) transfected by pCAGGS-hPrx I-HA-neo vector were detected by flow cytometry. *p < 0.05, **p < 0.01 compared to Mock. HCC cells; Huh7 and SK-HEP-1 cells. The data were repeated in at least three separate experiments and presented as mean ± SD.

In oncogene-mediated tumorigenicity, ROS are essential to activate critical signaling pathways and promote cellular proliferation [22, 23]. Prx I is a scavenger of ROS that belongs to the peroxidase family of antioxidant enzymes. Knockdown of Prx I in Huh7-H-ras^G12V^ cells and SK-HEP-1-H-ras^G12V^ cells significantly increased the intracellular ROS level (Figure 4A) and cell apoptosis (Figure 4B). Consistently, FACS analysis showed that H-ras^G12V^/Prx I^−/−^ double mutant mice had high levels of ROS and apoptosis, especially at 7 months (Figure 4C and 4D); Histological analysis showed that ROS-induced DNA damage and cell apoptosis were dramatically higher in liver tissues of H-ras^G12V^/Prx I^−/−^ double mutant mice at 7 months than H-ras^G12V^/WT mice (Figure 4E), whereas Prx I overexpression in SK-HEP-1 cells significantly suppressed intracellular ROS and cell apoptosis (Figure 4F). In summary, Prx I expression in the liver is able to protect hepatic carcinoma cells from ROS-induced cell death to promote hepatocarcinogenesis.

### Prx I regulated transcription factor FoxM1 and Nrf2 levels in H-ras^G12V^ induced hepatic tumors

**Figure 5:**
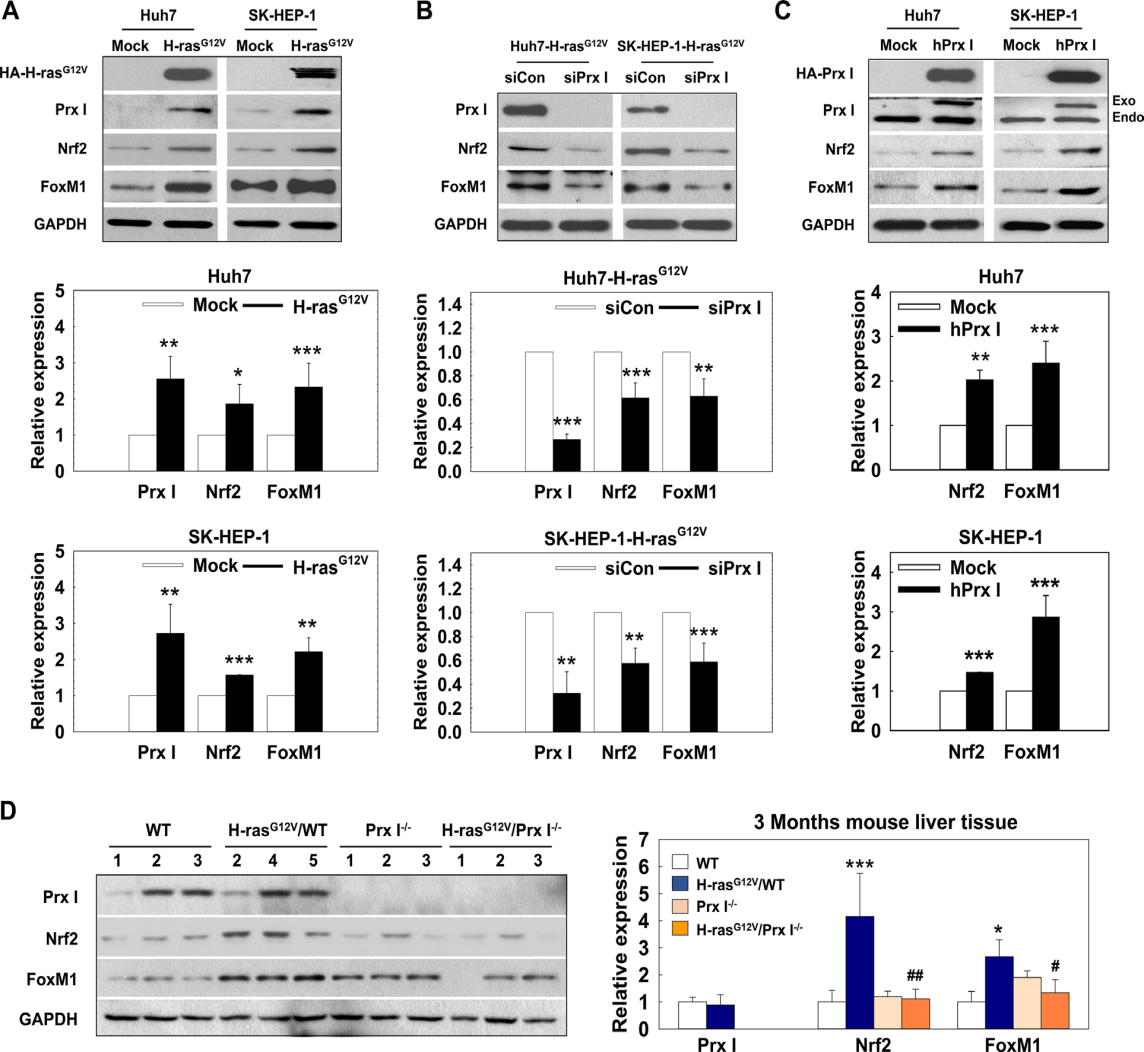
Prx I regulated transcription factor FoxM1 and Nrf2 levels in H-ras^G12V^-induced hepatic tumors (**A**–**D**). Prx I, Nrf2, FoxM1, and GAPDH expression levels were detected in HCC-Mock cells and HCC-H-ras^G12V^ cells (A); siPrx I-transfection HCC-H-ras^G12V^ cells (B); pCAGGS-hPrx I-HA-neo-transfected Prx I-HCC cells (C); and 3 months WT, Prx I^−/−^, H-ras^G12V^/WT, and H-ras^G12V^/Prx I^−/−^ mice liver tissues (D) by Western blotting. In (A, B and C), *p < 0.05, **p < 0.01, ***p < 0.001 compared to Mock, in D, *p < 0.05, ***p < 0.001 compared to WT and ^#^*p* < 0.05, ^##^*p* < 0.01 compared to H-ras^G12V^/WT. HCC cells; Huh7 and SK-HEP-1 cells. The data were repeated in at least three separate experiments.

Forkhead box protein M1 (FoxM1) and Nrf2 are both important transcription factors in carcinogenesis [24]. FoxM1 downregulates ROS levels by stimulating expression of ROS scavenger genes, such as MnSOD, catalase and PRDX3 [25]; the Nrf2-Prx I axis is important in human lung-cancer, prostate-cancer, colon-cancer, and breast-cancer development [26]. In our data, FoxM1 and Nrf2 significantly increased in HCC-H-ras^G12V^ cells, accompanied by increased Prx I expression (Figure 5A).

To determine the relationship between Prx I and transcription factors (FoxM1 and Nrf2), we performed Western blotting analysis in HCC-H-ras^G12V^ cells. The data showed that knockdown of Prx I dramatically down-regulated Nrf2 and FoxM1 expression (Figure 5B) and conversely, overexpression of Prx I in HCC cells up-regulated Nrf2 and FoxM1 protein levels (Figure 5C). Mouse-liver tissue samples also showed consistent data (Figure 5D). Taken together, these results suggested that Prx I and transcription factor (FoxM1 and Nrf2) expression closely correlate and Prx I positively upregulates FoxM1 and Nrf2 expression.

### Oncogene Ras activates the ERK/FoxM1/Nrf2/Prx I pathway to promote hepatocarcinogenesis

**Figure 6:**
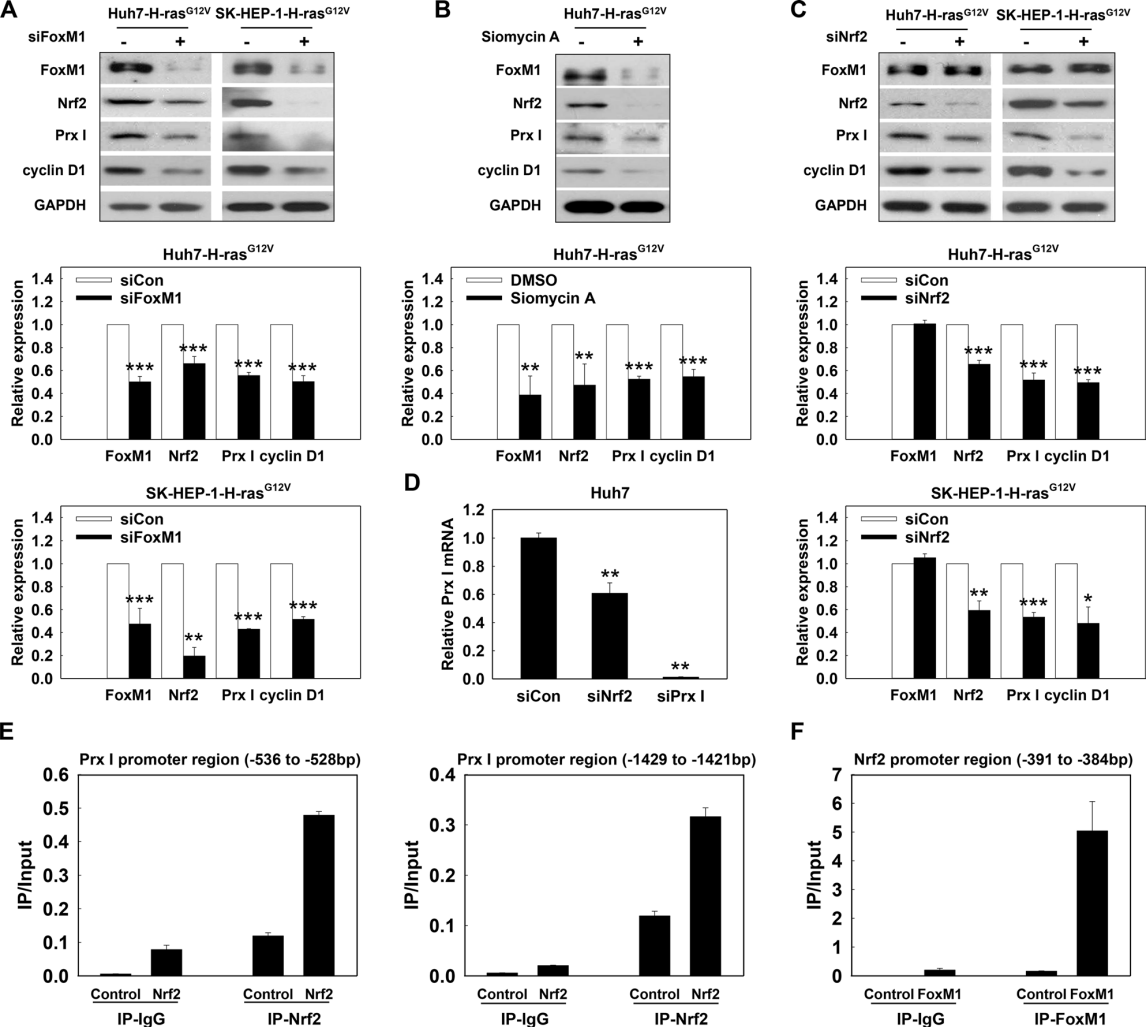
Oncogene Ras activated the ERK/FoxM1/Nrf2/Prx I pathway (**A**) Huh7-H-ras^G12V^ cells and SK-HEP-1-H-ras^G12V^ cells were transfected with siFoxM1 for 72 h; (**B**) Huh7-H-ras^G12V^ cells were treated with Siomycin A (10 μM) for 24 h. After incubation, the cell lysates were used to determine protein expression. (**C**) After HCC-H-ras^G12V^ cells transfected with siNrf2 for 72 h, the protein expression of FoxM1, Nrf2, Prx I, cyclin D1, and GAPDH were detected in the cell lysates. In A and C, *p < 0.05, **p < 0.01, ***p < 0.001 compared to siCon. In B, **p < 0.01, ***p < 0.001 compared to DMSO. (**D**) Prx I mRNA level was detected in Huh7 cells transfected with siRNA (scramble, Nrf2 or Prx I) for 48 h, **p < 0.01 compared to siCon. (**E** and **F**) CHIP assay was performed to detect the Nrf2 binding sites in the Prx I promoter region (–1429 to –1421), (–536 to –528) (E) or FoxM1 target site in the Nrf2 promoter region (–391 to –384) (F). HCC-H-ras^G12V^ cells; Huh7- H-ras^G12V^ and SK-HEP-1- H-ras^G12V^ cells. The data were repeated in at least three separate experiments.

Knockdown of FoxM1 by siRNA caused a reduction of Prx I, Nrf2 and cyclin D1 protein in Huh7-H-ras^G12V^ and SK-HEP-1-Hras^G12V^ cells (Figure 6A). Consistent results are shown in Huh7-H-ras^G12V^ cell treatment with Siomycin A, a potent specific inhibitor of FoxM1 (Figure 6B). However, knockdown of Nrf2 only down-regulated Prx I and cyclin D1 protein levels, and did not affect FoxM1 expression (Figure 6C). CHIP assay showed that in Huh7 cell, Nrf2 binds to the Prx I promoter region (–536 to –528) and (–1429 to –1421) to regulate Prx I gene expression. RT-PCR results, consistent with CHIP assay, showed that Prx I mRNA levels were significantly reduced by knockdown of Nrf2 (Figure 6D and 6E).

**Figure 7:**
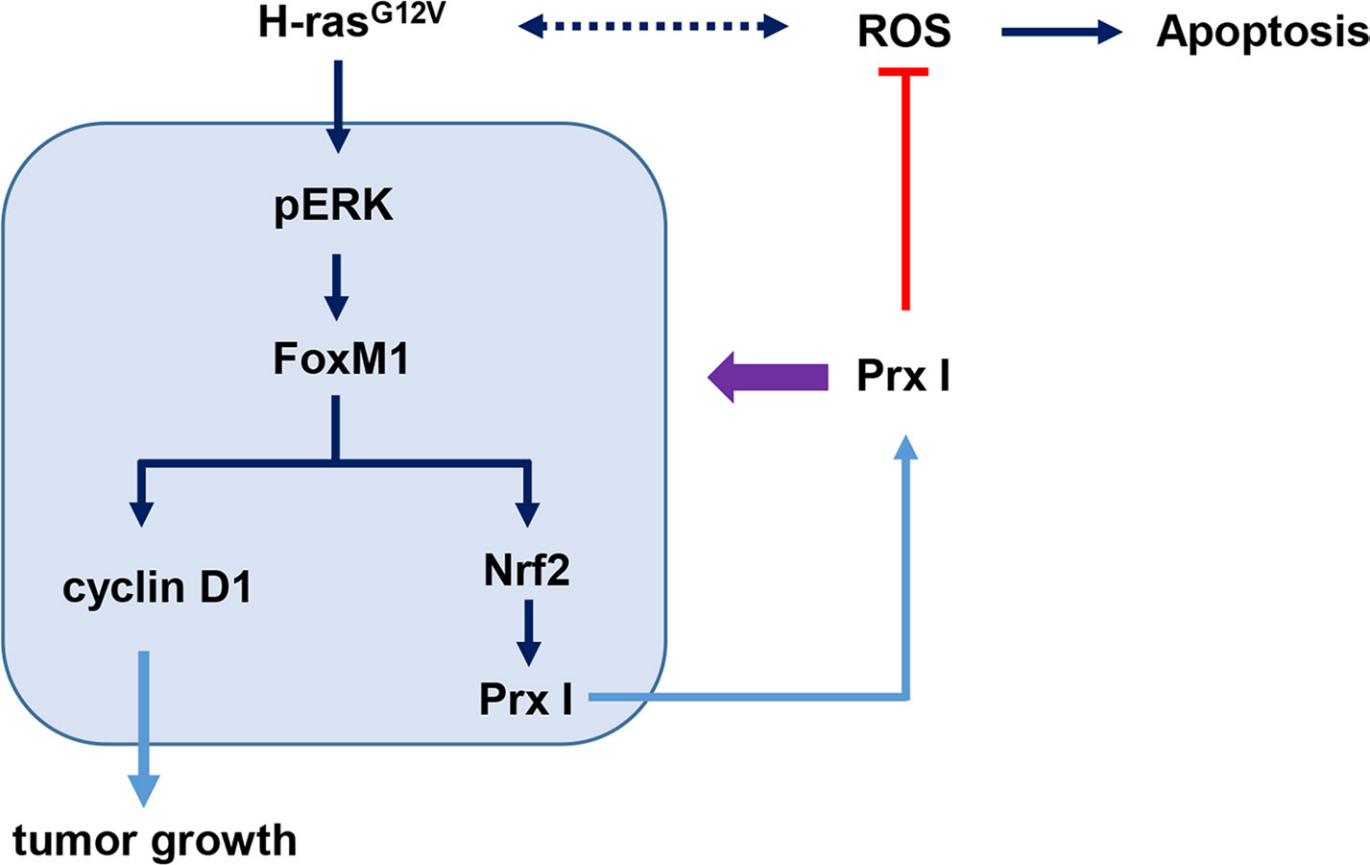
Schematic illustration of Prx I function in oncogenic Ras-induced liver tumorigenesis H-ras^G12V^ enhanced intracellular ROS levels and promoted downstream pERK/FoxM1 pathway. Transcription factor FoxM1 targeted the cell-cycle-related protein cyclin D1 and Nrf2 expression. Then Nrf2 targeted Prx I promoter to regulate Prx I expression. Overexpressing Prx I positively regulated the ERK/FoxM1/Nrf2 pathway and suppressed ROS-induced cell death to promote hepatic tumorigenesis. However, knockdown Prx I failed to scavenge ROS-induced cell death, which inhibited the tumorigenesis.

To investigate whether FoxM1 controls Nrf2 at the transcriptional level, we used silico prediction of transcription factors binding to an Nrf2 promoter, and found that the FoxM1B/FoxA binding site (TTTGTTTGTTTG) was located at the human Nrf2 promoter –391 to –384, upstream of the transcriptional start site. CHIP analysis indicated that FoxM1 binds to the FoxM1 binding site of the Nrf2 promoter region (Figure 6F) and revealed that Nrf2 is a transcription target of FoxM1. Taken together, H-ras^G12V^ modulates hepatic tumorigenesis through regulation of the ERK/FoxM1/Nrf2/Prx I pathway, and Prx I is able to facilitate the ERK/FoxM1/Nrf2 pathway by forming a positive feedback loop between Prx I and the ERK/FoxM1/Nrf2 pathway (Figure 7).

## DISCUSSION

Prx I is an important antioxidant enzyme in tumor development. In the current study, we demonstrated that Prx I has an essential role in H-ras-induced hepatic cellular carcinoma. Prx I deficiency in HCC-H-ras^G12V^ cells and H-ras^G12V^/Prx I^−/−^ double mutant mice led to reduced HCC-H-ras^G12V^ cell-colony formation and decreased hepatic-tumor numbers and sizes, respectively. Prx I protected tumor cells from ROS-induced cell death and positively regulated the H-ras^G12V^-induced ERK/FoxM1/Nrf2 pathway to promote tumorigenesis.

Results from HCC patients' sample analysis showed that Prx I is a diagnostic and prognostic biomarker of HCC, and high levels of Prx I mRNA in tumor tissue aligned with a shorter overall survival time [27]. Moreover, increased Prx I expression positively correlated with endothelial tumor growth, vessel density, and tumor size [28]. Decreased expression of Prx I in HepG2 cells led to changes in cell morphology and delayed cell growth [29]. These data were consistent with our present data. In the HCC-patient tumor region, Prx I expression was higher than in the non-tumor region. In the H-ras^G12V^-induced HCC mouse model, hepatic tumor growth was time dependent. Interestingly, Prx I expression also increased in H-ras^G12V^/WT Tg mice in a time-dependent manner. Knockdown of Prx I expression in HCC-H-ras^G12V^ cells dramatically suppressed cell proliferation and colony formation; Prx I knockout in H-ras^G12V^ Tg mice significantly decreased tumor size and numbers. At 7 months H-ras^G12V^ Tg mice markedly decreased in body weight and increased in liver weight. However, Prx I knockout in Hras^G12V^ Tg mice significantly recovered the body- and liver-weight changes (Supplementary Figure S1). Taken together, Prx I contributes to hepatic-tumor development.

ROS are well known to activate human cancers and their function in cells might be as a double-edged sword. A moderate increase of ROS may promote cell proliferation and survival by triggering a redox-adaptation response, leading to enhanced cellular antioxidant capability to help cells survive under certain levels of oxidative stress [30–33]. However, when ROS are increased to a certain level, they may overwhelm the antioxidant capacity of the cell and trigger cell death [34]. So when K-ras-induced ROS are moderate, they can help in tumorigenicity [23]. In addition, recently it was reported that oncogenic K-ras-activated expression of antioxidant genes via Nrf2 mediated an unusual metabolic pathway of glutamine to generate NADPH, which led to low levels of ROS in pancreatic pre-neoplastic cells. The low level of ROS promotes carcinogenesis in K-ras-mutated pancreatic ductal adenocarcinoma (PDAC) [35]. However, a further increase of ROS may break the antioxidant system and elevating cellular ROS above the threshold level would lead to cell death [22, 34, 36]. Thus, modulating the ROS-induced signaling pathway is important for tumorigenesis.

Prx I is an antioxidant enzyme activated by oxidative stress that regulates multiple signals in different cancers. In breast cancer, Prx I suppressed ROS-induced AKT phosphorylation to inhibit cell proliferation [37]; In lung cancer, Prx I suppressed the ROS-induced ERK/cyclin D1 pathway to inhibit tumor growth [11]; In pancreatic cancer, Prx I modulated P38 activation to promote cancer-cell invasion [38]; In liver cancer, Prx I is an immediate and sensitive marker of oxidative stress. Inhibition of Prx I cysteine activation in hepatocellular carcinoma cells dramatically increased intracellular ROS levels, leading to enhanced cytotoxicity in HCC cells [36, 39]. However, the mechanism of Prx I in HCC was still unclear.

In this study, we used an H-ras^G12V^-induced HCC model to identify Prx I function. We found that the increased ROS level in H-ras mutant form was under the toxic thresholds that aid in cancer-cell proliferation and survival. When we knocked down or overexpressed the antioxidant Prx I, the redox balance was broken. A small change in the ROS level over the threshold level loses the ability to adapt to oxidative stress, and thus increases cancer cell death. Thus, over time, small changes in ROS and apoptosis between H-ras^G12V^ Tg mice and H-ras^G12V^/Prx I^−/−^ double mutant mice induced different rates of carcinogenesis.

FoxM1 is a typical proliferation-associated transcription factor overexpressed in numerous cancer-cell lines and human cancers [40–42], implicated in cell migration, invasion, angiogenesis, and metastasis [43]. Nrf2, also an important transcription factor activated by oxidative stress in tumor development [44–46], controls cell fate by transcriptional upregulation of antioxidant enzyme genes to resist oxidative stress in cancer cells. Prx I is an important target gene of Nrf2. The Nrf2–Prx I axis links with tumor biology, enhancing the drug efficacy of cancer therapy and serving as a fruitful target of cancer prognosis [26]. MEK-ERK activation has been shown to be one of the major pathways resulting in the activation of Nrf2 and induction of Nrf2 downstream targets, including phase II detoxifying/antioxidant genes in response to oxidative stress [47]. In this study, ERK phosphorylation was significantly induced in H-Ras^G12V^ HCC cells compared with mock cells. In addition, Nrf2 also significantly increased in accordance with increased expression of Prx I. Furthermore, Nrf2 bound to the Prx I promoter region to regulate Prx I gene expression, as reported in lung cancer cells [11, 26]. Like Nrf2, FoxM1 also significantly increased in accordance with increased expression of Prx I in H-Ras^G12V^ HCC cells. It is interesting to note that FoxM1 activated Nrf2 via its binding to Nrf2 promoter region, not to Prx I. These data suggest that H-ras^G12V^ induced ERK phosphorylation increased transcriptional expression of Prx I through FoxM1/Nrf2 signaling.

ROS increment by Ras activation was shown to activate expression of pERK, FoxM1 and Nrf2. However, when ROS level was more increased with Prx I knockdown or deletion in H-Ras^G12V^ HCC cells, expression levels of FoxM1 and Nrf2 decreased. Why does increased ROS lead to a decrease in FoxM1 and Nrf2 levels? Previously it was reported that FoxM1 and Nrf2 are pERK substrates [41, 47, 48]. In this experiment, the activation of FoxM1 and Nrf2 was depended on the ERK activity. Knockdown or deletion of Prx I down-regulated the ERK activity. Thus the expression of FoxM1 and Nrf2 was reduced even though the ROS level increased. In addition, overexpression of Prx I in HCC cell lines enhanced pERK, then the expression of FoxM1 and Nrf2, although it decreased the ROS level (Figure 3C). However, further studies are needed to understand the reason why increased ROS lead to a decrease in FoxM1 and Nrf2 levels.

The conclusion is that Prx I positively regulates the H-ras^G12V^-induced ERK/FoxM1/Nrf2 pathway, and suppresses ROS-induced cell death to promote hepatocarcinogenesis. This outcome can be applied when studying a novel mechanism for HCC prognosis and therapy.

## MATERIALS AND METHODS

### Animal models

H-ras^G12V^ transgenic and Prx I knockout mice were previously described [11, 49]. Hras^G12V^/Prx I^−/−^ double mutant mice were generated by crossing H-ras^G12V^ Tg mice with Prx I knockout mice. The mouse room was maintained at a constant temperature of 22 ± 1°C, humidity of 55 ± 10%, and 12 hr light/dark cycle. We used genotyping primers, as previously reported [11, 49]. All animal procedures were conducted in accordance with the guidelines of the Insitutional Animal Care and Use Committee, Korea Research Institute of Bioscience and Biotechnology.

### Patient tissue sample

Patient HCC tissues were collected from Chonbuk National University hospital. All study patients provided informed consent prior to surgery.

### Cell culture, transfection and treatment

Human HCC cell lines—Huh7, SK-HEP-1, HepG2, Hep3B, PLC/PRF/5 and SNU449 (ATCC, Manassas, VA, USA)—were maintained in Dulbecco's modified Eagle's medium (DMEM), Roswell Park Memorial Institute 1640 medium (RPMI 1640) containing 10% fetal bovine serum (FBS), penicillin (100 U/ml), and streptomycin (100 mg/ml). Cells were incubated in a humidified atmosphere with 37°C, 5% CO_2_ and 95% air. The Huh7- and SK-HEP-1-H-ras^G12V^ mutant cell lines were generated by transfection. The transfection vector pCAG-HA-H-ras^G12V^-neo was constructed as follows: The coding sequences for mutated H-ras (H-ras^G12V^) were inserted by PCR cloning into the EcoRI site of the pCAG-HA-neo vector and confirmed by restriction enzyme mapping and DNA sequencing. Huh7 and SK-HEP-1 cells were plated in 6-well culture plates for 24 h prior to transfection. Cells were transfected with 3 μg of a pCAG-HA-H-ras^G12V^-neo construct using a Lipofectamine LTX reagent (Invitrogen, Waltham, MA, USA) according to the manufacturer's instructions. After 48 h, cells were trypsinized and plated in a medium containing 400 μg/ml neomycin (G418). Following selection for 2 weeks, total populations of neomycin-resistant cells were pooled and single-cells sorted into 96-well plates with a growth medium containing 400 μg/ml of neomycin. Sorted single cells were grown under selection for an additional 2 weeks and expanded into stable cell lines. Candidate clones were analyzed by immunoblotting using a specific H-ras (Santa Cruz Biotechnology, Dallas, TX, USA) or HA (Roche, Basel, Switzerland) antibody. Transfection of pCAGGS-hPrx I-HA-neo vectors or siRNA (Prx I, Nrf2, and FoxM1) to overexpress Prx I or knockdown their proteins level was described in previous papers [11, 49]. Huh7-H-ras^G12V^ cells were treated with 10 uM Siomycin A, FoxM1 inhibitor (Calbiochem, Billerica, MA, USA) for 24 h.

### Western blotting

Western blotting was performed as described previously [11]. Liver tissues and cell lysates were put in a lysis buffer (20 mM HEPES, 150 mL NaCl, 2 mM EGTA, 1 mM EDTA, 20 mM glycerol phosphate, 1% Triton X-100 and 10% glycerol) with protease (sigma, St Louise, MO) and a phosphatase-inhibitor cocktail (Roche, Basel, Switzerland). Protein samples were extracted by an SDS-PAGE and transferred to nitrocellulose membranes (Millipore, Bellerica, MA, USA). The membranes were primarily blotted with primary antibodies against Prx I, PCNA, Nrf2 (Abcam, Cambridge, UK); HA (Roche, Basel, Switzerland); GAPDH, FoxM1 (Abfrontier, Seoul, Korea); pERK, pP38, pJNK, cyclin D1 (Cell Signaling Technology, Danvers, MA, USA); and H-ras (Santa Cruz Biotechnology, Dallas, TX, USA).

### Cell-proliferation assay

1,000 cells per well were seeded in a 96-well cell-culture plate. After incubating for 12 h, cells were transfected with siCon or siPrx I, then cultured for 5 days. Cell Counting Kit-8 (CCK8, Dojindo Molecular Technologies, Rockville, MD, USA) was added to the plate, incubated at 37°C for 3 h, with absorbance measured by a microplate reader (VERSAmaxTM, Madison, NC, USA) at a wavelength of 450 nm.

### Colony-formation assay

5,000 cells were pretreated with siRNA (Scramble or Prx I) for 24 h, suspended in 1 ml DMEM containing 0.3% agar and plated on 0.6% agar in a cell-growth medium, then put in a 37°C and 5% CO_2_ incubator for 2 weeks. Colonies were observed under an inverted phase-contrast microscope. Data represent at least triplicate independent wells.

### Immunohistochemistry

Liver tissues were fixed in 10% paraformaldehyde and embedded in paraffin. Sections (3 μm) were stained with hematoxylin and eosin (H&E) or subjected to immunohistochemistry. Additional details were performed as previously described [50]. The primary antibodies used were Prx I, PCNA (Abcam, Cambridge, UK); pERK (Cell Signaling Technology, Danvers, MA, USA); and 8-oxoguanine (8-oxo-dG; Millipore, Bellerica, MA, USA). The TUNEL assay was performed using the ApopTag Peroxidase Kit (S-7100; Millipore).

### Detection of ROS and apoptosis by flow cytometry

To measure the intracellular ROS and cell apoptosis, cells were stained with 10 uM 5, 6-chloromethyl-2′, 7′-dichlorodihydrofluorescein diacetate (CM-H_2_DCFDA; Invitrogen, Carlsbad, CA, USA) and Annexin V (BD Biosciences, Franklin Lakes, NJ, USA) for 15 min at 37°C, and washed with PBS twice by centrifugation at 600 g for 3 min. The cells resuspended with PBS and were analyzed by flow cytometry on a FACSCalibur instrument (BD Biosciences).

### CHIP assay

This experiment was performed using the chromatin immunoprecipitation assay (CHIP assay) kit (Cell Signaling Technology, Danvers, MA, USA) according to the supplied protocol. Antibodies Nrf2, FoxM1, and IgG were added to sonicated Huh7 cell lysates for immunoprecipitation at 4°C with rotation overnight; we then added Chip-Grade Protein G Agarose Beads to pull down the protein-DNA complex, and amplified the binding DNA by qPCR, using the Nrf2 binding regions of the human Prx I promoter (–1429 to –1421) or (–536 to -528): 5′-tcactgcaacctctgcctcc-3′ and 5′-gatcgagaccatcctggcca-3′; 5′-ttgccttaccgtgtgggtctg-3′ and 5′-actcctaaacttacgccagagg-3′. FoxM1 binding human Nrf2 promoter regions: 5′-agctcctacaccaacgcctt-3′ and 5′-cggcgttcagtttgccctcc-3′. The nonbinding position (control) of the human Prx I promoter was 5′-gccaacatgatgaaacctcgtc-3′ and 5′-ggtgttggcttactgcaacctcc-3′. A nonimmunoprecipitated chromation fragment was used as an input control.

### Statistical analysis

The data were analyzed using SigmaPlot 13 software for Student's *t*-test, a value of *p* less than 0.05 was considered to be statistically significant.

## SUPPLEMENTARY MATERIALS FIGURES



## Abbreviations

Ade: adenoma
Ca: carcinoma
Dys: dysplasia
hPrx I: human Prx I
N: non-tumor
Prx I: peroxiredoxin I
siCon: scramble siRNA
T: tumor
3 M: 3 months
7 M: 7 months.
