# Supplementary material for: Peroxiredoxin I is important for cancer-cell survival in Ras-induced hepatic tumorigenesis
Source: Oncotarget. 2016 Aug 10;7(42):68044–56. doi: 10.18632/oncotarget.11172 (PMC5356538; doi:10.18632/oncotarget.11172)
Supplement: Supplementary file 1 [file oncotarget-07-68044-s001.pdf]

Peroxiredoxin I is important for cancer-cell survival in Ras-induced Hepatic tumorigenesis

Supplementary Materials

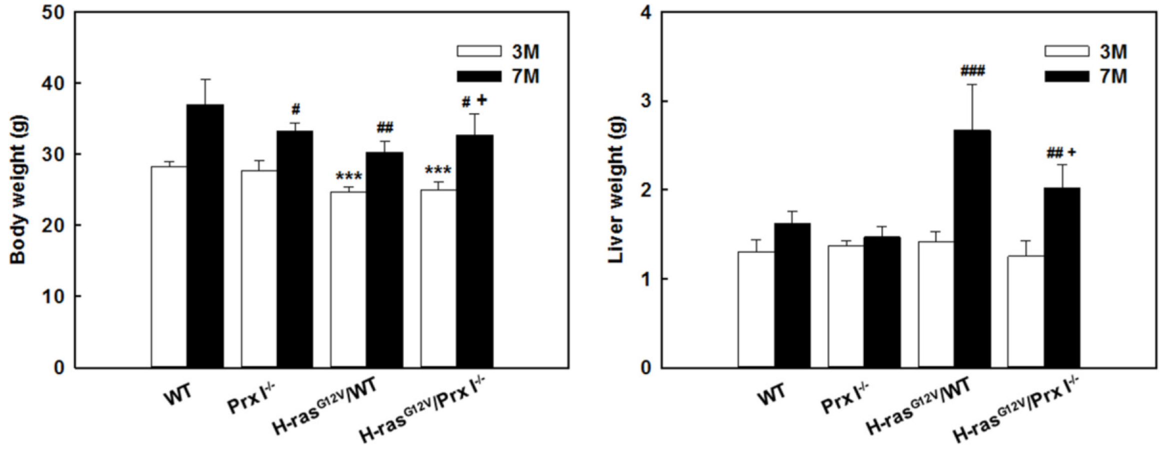

Supplementary Figure S1: The effect of Prx I knockout on body weight and liver weight of H-ras^{G12V} transgenic mouse. The body weight and liver weight of WT, Prx I$^{-/-}$, H-ras^{G12V}/WT, and H-ras^{G12V}/Prx I$^{-/-}$ mice at indicated time points are shown. 3 M, 3 months; 7 M, 7 months. $^{***}p < 0.001$ compared to WT mice at the age of 3 months, $^{\#}p < 0.05$, $^{\#\#}p < 0.01$, $^{\#\#\#}p < 0.001$ compared to WT mice at the age of 7 months, and $^{+}p < 0.05$ compared to H-ras^{G12V}/WT mice at the age of 7 months. The data were repeated in at least three separate experiments and presented as mean ± *SD*.

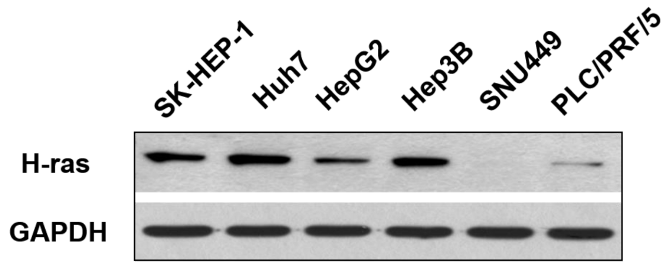

Supplementary Figure S2: Western blotting analysis of H-ras expression in HCC cells. The data were repeated in at least three separate experiments.
